# Comparative Effects of Processed Soybean Protein Sources on Growth, Serum Immune and Endocrine Profiles, and Colonic Microbiota in Weaned Piglets

**DOI:** 10.3390/ani16172722

**Published:** 2026-09-01

**Authors:** Haoliang Chai, Dexin Zhao, Hanyang Wang, Linfeng Zhou, Xinping Diao, Liangmei Xu, Hongzhi Wu

**Affiliations:** 1 Tropical Crops Genetic Resources Institute, Chinese Academy of Tropical Agricultural Sciences, Haikou 571101, China; 2 College of Animal Science and Technology, Northeast Agricultural University, Harbin 150030, China

**Keywords:** weaned piglets, processed soybean protein, nutrient digestibility, serum immunity, colonic microbiota

## Abstract

Weaning stress and antinutritional components in conventional soybean meal may impair intestinal homeostasis and growth in nursery piglets. This study compared five soybean protein sources: conventional soybean meal (SBM), rapidly expanded soybean meal (RSB), fermented soybean meal (FSBM), hydrolyzed soybean protein (HP), and extruded full-fat soybean (ESB). Two concurrent 28-day trials used 120 newly weaned piglets, with 60 piglets per trial allocated to five treatments (six pens per treatment and two piglets per pen): a single-diet trial and a dual-choice feed-preference trial. Growth, feed preference, crude protein digestibility, serum biomarkers, and colonic microbiota were evaluated. HP showed the highest in vitro and apparent crude protein digestibility and increased final body weight and average daily gain relative to SBM and RSB (*p* < 0.05). Compared with SBM, HP increased serum IgA, IL-2, GH, and IGF-1 and decreased TNF-α (*p* < 0.05). Serum antioxidant indices and microbial alpha diversity did not differ among treatments (*p* > 0.05). Descriptive genus-level analyses indicated a higher relative abundance of Lactobacillus in the HP and FSBM groups. Overall, HP produced the most consistent improvements in protein digestibility and selected growth and serum outcomes under the conditions of this study.

## Simple Summary

Conventional soybean meal is the principal plant protein source in piglet diets, but its antinutritional and antigenic components may impair intestinal function in newly weaned piglets. These components can contribute to reduced feed intake, diarrhea, and impaired growth during the nursery phase. Although extrusion, fermentation, and enzymatic hydrolysis are widely used to improve soybean nutritional quality, most studies have evaluated only a limited set of outcomes. This 28-day study compared five commercially available soybean protein ingredients using growth, feed preference, protein digestibility, serum biomarkers, and colonic microbiota. Hydrolyzed soybean protein contained no detectable glycinin and the lowest measured concentration of major antigenic proteins, showed the highest crude protein digestibility, and was associated with higher serum IgA, GH, and IGF-1 concentrations, lower TNF-α concentrations, and greater weight gain. Fermented and rapidly expanded soybean meals produced more modest responses. Feed preference did not differ among treatments. Alpha diversity also did not differ significantly, whereas the relative abundance of Lactobacillus was higher in the hydrolyzed and fermented soybean protein groups in the descriptive genus-level analysis. These results provide comparative evidence to guide the selection of processed soybean proteins for nursery pig diets.

## 1. Introduction

Early weaning improves sow reproductive efficiency but exposes piglets to abrupt dietary, environmental, and social changes while their digestive and immune systems remain immature [1,2]. The resulting reduction in feed intake and digestive capacity can increase the risk of intestinal dysfunction, diarrhea, and impaired growth [3,4,5]. Nutritional strategies that support adaptation during this period remain important in antibiotic-reduced production systems. However, processed soybean proteins are often evaluated using isolated outcomes, and direct comparisons integrating growth, feeding behavior, nutrient utilization, systemic physiology, and gut microbiota are limited.

Soybean meal has a favorable amino acid profile but contains trypsin inhibitors, glycinin, β-conglycinin, lectins, and oligosaccharides [6,7]. Newly weaned piglets digest complex plant proteins inefficiently, allowing undigested protein to enter the hindgut and support proteolytic fermentation [3,4,8]. Glycinin and β-conglycinin may elicit hypersensitivity, trypsin inhibitors reduce proteolysis, and oligosaccharides can increase osmotic and fermentative pressure [7,9,10]. Reducing these components while maintaining balanced dietary nutrient supply may therefore improve protein utilization during weaning.

Processing can alter soybean protein structure and reduce antinutritional factors [11]. Extrusion uses heat, pressure, and shear to inactivate heat-labile compounds and modify protein conformation. Fermentation can degrade antigenic proteins and generate peptides, organic acids, and microbial metabolites [10,12]. Enzymatic hydrolysis produces smaller peptides and free amino acids that may be more accessible to the immature digestive tract [11]. Hydrolyzed soybean protein (HP) is therefore of interest, but its effects have rarely been compared with several commercial alternatives under the same experimental conditions.

Negative-pressure explosive expansion, referred to here as rapid expansion, subjects soybean material to pressure followed by rapid decompression. The abrupt pressure release disrupts the compact matrix and increases porosity, which may improve enzymatic accessibility. The RSB product used in this study contained less glycinin and β-conglycinin than conventional SBM (Table 1); this compositional comparison is specific to the tested commercial batches and should not be generalized to all rapidly expanded products. We compared SBM, RSB, FSBM, HP, and ESB using growth performance, feed preference, crude protein digestibility, serum biochemical, immune, antioxidant, and endocrine indices, and colonic microbial composition. We hypothesized that processing-related reductions in antigenic proteins would be associated with improved protein digestibility and physiological adaptation.

## 2. Materials and Methods

### 2.1. Experimental Diets and Raw Materials

The five experimental diets differed in their principal soybean protein source: conventional soybean meal (SBM; control), rapidly expanded soybean meal (RSB), fermented soybean meal (FSBM), hydrolyzed soybean protein (HP), or extruded full-fat soybean (ESB). SBM, FSBM, and ESB were provided by Harbin Pufan Feed Co., Ltd. (Harbin, China); RSB was purchased from Hangzhou Xiangbao Feed Co., Ltd. (Hangzhou, China); and HP was purchased from Hamlet Protein A/S (Horsens, Denmark). All diets were manufactured by Harbin Pufan Feed Co., Ltd. Diets were formulated to be comparable in major nutrients and isoenergetic, with calculated digestible energy ranging from 14.10 to 14.20 MJ/kg, and were balanced for standardized ileal digestible lysine, methionine, threonine, and tryptophan. Analyzed crude protein ranged from 17.35% to 17.62% (Table 2). 

Trypsin inhibitor, glycinin, and β-conglycinin concentrations in the five soybean protein products were determined using commercial ELISA kits for Kunitz trypsin inhibitor (KTI; cat. no. mlsh8347), soybean 11S globulin/glycinin (cat. no. mlsh8334), and β-conglycinin (cat. no. mlsh8337), respectively (Shanghai Enzyme-linked Biotechnology Co., Ltd., Shanghai, China), according to the manufacturer’s instructions. Samples were homogenized in phosphate-buffered saline (PBS; pH 7.4), and supernatants were collected after centrifugation at 3000× *g* for 15 min at 4 °C. Optical density was measured at 450 nm using a Model 680 microplate reader (Bio-Rad Laboratories, Hercules, CA, USA), and concentrations were calculated from kit standard curves. Results are reported as mg/g of ingredient. Each ingredient was analyzed in three technical replicates, and the mean was used for descriptive reporting.

### 2.2. Animal Arrangement and Feeding Regime

A total of 120 healthy, newly weaned Tunchang Black piglets (barrows and females, 1:1; initial body weight, 5.86 ± 0.11 kg) were used in two concurrent 28-day trials. Each trial included 60 piglets allocated to five treatment groups, with six replicate pens per group and two piglets per pen. Piglets were blocked by body weight and randomly assigned within blocks, with sex balanced across groups. In the single-diet trial, the five groups received the SBM, RSB, FSBM, HP, or ESB diet, respectively; this trial generated the growth, serum, digestibility, and microbiota data. In the dual-choice trial, SBM served as the common reference diet and the four processed soybean diets were evaluated as test diets; this trial generated only the feed-preference data. The pen was the experimental unit in both trials. The Tunchang Black pig was selected because it is an economically important indigenous breed in Hainan Province and evidence concerning processed soybean proteins in local Chinese breeds remains limited. Breed-related differences in growth, digestive physiology, and microbiota may limit direct extrapolation to commercial crossbred pigs. Piglets were housed at 24–28 °C and 60–70% relative humidity under a 12-h light:12-h dark cycle, with free access to feed and water. Pens were randomly distributed within the barn, and all piglets completed a 7-day adaptation period on the basal diet before the trials.

All animal procedures described in this study were approved by the Institutional Animal Care and Use Committee of the Chinese Academy of Tropical Agricultural Sciences (approval number: CATAS20250801-09) and conducted in accordance with local legislation and institutional requirements.

### 2.3. Determination of Feed Preference and Preference Index

A dual-choice feeding assay was used to determine feed preference (FP, %) and the feed preference index (FPI). The SBM diet served as the common reference diet, and the RSB, FSBM, HP, and ESB diets were evaluated separately as test diets. Each pen was equipped with two symmetrically positioned troughs, one containing the reference diet and the other containing the corresponding test diet. Trough positions were alternated daily to minimize positional bias. Feed offered and residual feed from each trough were weighed daily. Cumulative pen-level intake over the 28-day trial was used to calculate FP and FPI. Each feeding comparison comprised six replicate pens, and the pen was the experimental unit.

FP (%) = 100 × (cumulative test-diet intake)/(cumulative test-diet intake + cumulative reference-diet intake)

FPI = cumulative test-diet intake/cumulative reference-diet intake

Thus, FP values above 50% and FPI values above 1.0 indicated a preference for the test diet relative to the SBM reference diet. Each treatment combination was replicated in six pens, and pen-level cumulative intake data were used for statistical analysis.

### 2.4. Sample Collection Protocols

After 28 days, piglets in the single-diet trial were deprived of feed for 12 h with free access to water. One piglet with body weight closest to the pen mean was selected from each pen (*n* = 6 per treatment) for blood collection and euthanasia. Blood was collected from the anterior vena cava into tubes without anticoagulant, allowed to clot at room temperature, and centrifuged to obtain serum, which was stored at −80 °C. Colonic digesta from the same piglet was aseptically collected, frozen in liquid nitrogen, and stored at −80 °C. Thus, serum analyses used six biological samples per treatment. Six colonic samples per treatment entered DNA quality control; four samples per treatment met the predefined criteria and were sequenced.

### 2.5. Growth Performance Measurements

Individual body weight was recorded at the beginning and end of the trial to calculate initial body weight (IBW), final body weight (FBW), and average daily gain (ADG). Feed offered and residual feed were recorded by pen to calculate average daily feed intake (ADFI) and feed conversion ratio (FCR). The pen, rather than the individual piglet, was the experimental unit for all growth outcomes.

### 2.6. In Vitro and Apparent Crude Protein Digestibility

In vitro crude protein digestibility was determined using a two-step simulated gastrointestinal digestion procedure. Samples were incubated with pepsin at pH 2.0 and 39 °C for 2 h, adjusted to neutral pH, and then incubated with pancreatin at 39 °C for 4 h. After digestion was stopped, the residue was filtered, dried, and analyzed for crude protein by the Kjeldahl method. In vitro digestibility was calculated from the difference between initial and residual crude protein.

Apparent total-tract crude protein digestibility was determined using acid-insoluble ash (AIA) as an internal marker. The AIA concentrations in the diets and feces were determined according to ISO 5985:2002 [13]. Briefly, the samples were incinerated to remove organic matter, and the resulting ash was treated with hydrochloric acid. The acid-insoluble residue was collected by filtration through ash-free filter paper, washed, dried, re-incinerated, and weighed to a constant mass. Apparent digestibility (%) was calculated as 100 − [100 × (AIA in feed/AIA in feces) × (crude protein in feces/crude protein in feed)]. Feces were collected by rectal stimulation on days 21, 24, and 27. Approximately 100 g was collected at each sampling, and samples were pooled within pen. Urine, hair, and other contaminants were excluded. Samples were dried, ground, and analyzed for crude protein using the Kjeldahl method. The pen-level pooled sample was the experimental unit.

### 2.7. Comprehensive Serum Parameter Detection

Serum samples were thawed on ice. Immunoglobulin A (IgA; kit cat. no. H108-1-2), immunoglobulin G (IgG; H109-1-2), immunoglobulin M (IgM; H110-1-2), interleukin-2 (IL-2; H002), tumor necrosis factor-α (TNF-α; H052), growth hormone (GH; H101), ghrelin (Ghr; H191), leptin (Lep; H104), cholecystokinin (CCK; H128), and insulin-like growth factor-1 (IGF-1; H095) were measured using commercial porcine ELISA kits (Nanjing Jiancheng Bioengineering Institute, Nanjing, China) according to the manufacturer’s instructions. Glutathione peroxidase (GSH-Px; kit cat. no. A005-1), superoxide dismutase (SOD; A001-3), catalase (CAT; A007-1-1), total antioxidant capacity (T-AOC; A015-2-1), and malondialdehyde (MDA; A003-1-2) were measured using spectrophotometric kits (Nanjing Jiancheng Bioengineering Institute, Nanjing, China). Total protein (TP; A045-2), albumin (ALB; A028-2), globulin (GLB; A029-2), triglycerides (TG; A110-1), total cholesterol (TC; A111-1), blood urea nitrogen (BUN; C013-2-1), high-density lipoprotein (HDL; A112-1), and low-density lipoprotein (LDL; A113-1) were measured with an automated clinical chemistry analyzer using matched commercial reagents (Nanjing Jiancheng Bioengineering Institute, Nanjing, China). Samples were assayed in duplicate, and the mean was used for analysis. Quality-control samples were included in each batch. Based on replicate quality-control measurements performed in the present study, the intra- and inter-assay coefficients of variation for all ELISA, spectrophotometric, and biochemical assays were below 5.0%, indicating acceptable analytical precision.

### 2.8. Colonic Microbiota High-Throughput Sequencing Analysis

Microbial DNA was extracted from 30 frozen colonic digesta samples using the DNeasy PowerSoil DNA Extraction Kit according to the manufacturer’s protocol. Concentration and purity were assessed with a NanoDrop 2000 spectrophotometer (Thermo Scientific, Waltham, MA, USA), and integrity was evaluated by 1% agarose gel electrophoresis. Prespecified criteria were DNA concentration ≥20 ng/μL, OD260/280 of 1.8–2.0, and an intact band without marked smearing. Two samples per treatment failed these criteria because of degradation or low yield; consequently, four samples per treatment (20 total) were sequenced. The V3–V4 region of the bacterial 16S rRNA gene was amplified with universal primers. Blank PCR controls were included. Amplicons were purified, quantified by PicoGreen, pooled at equal molarity, and sequenced as paired-end 2 × 250-bp reads on an Illumina NovaSeq platform. Raw paired-end reads were demultiplexed, and primer sequences were removed before import into QIIME 2 (version 2022.2) [14]. Sequence-quality profiles were inspected before denoising. Quality filtering, denoising, paired-end read merging, and chimera removal were performed using the DADA2 denoise-paired plugin [15]. No additional bases were removed from the 5′ ends of either the forward or reverse reads, and reads were not truncated at the 3′ ends (trim-left-f = 0, trim-left-r = 0, trunc-len-f = 0, and trunc-len-r = 0). Chimeric sequences were identified and removed using the consensus method. The resulting exact sequence variants were retained as amplicon sequence variants (ASVs).

### 2.9. Statistical Analysis

The pen was the experimental unit for growth, feed intake, feed preference, and digestibility outcomes (*n* = 6 pens per treatment). For serum outcomes, one piglet sampled from each pen represented that pen (*n* = 6 per treatment). Microbiota analyses included four qualified samples from four independent pens per treatment. Normality and homogeneity of variance were evaluated using the Shapiro–Wilk and Levene tests, respectively. Data meeting parametric assumptions were analyzed by one-way analysis of variance followed by Duncan’s multiple-range test. Analyses were conducted in SPSS Statistics version 24.0 (IBM Corp., Armonk, NY, USA). Results are presented as means with the standard error of the mean (SEM), and *p* < 0.05 was considered significant. Because a valid PERMANOVA output and multiple-comparison-adjusted differential-abundance analysis were unavailable, microbiota ordinations and relative-abundance graphics were treated as exploratory and no inferential *p* or *q* values were assigned to these visual patterns.

## 3. Results

### 3.1. Growth Performance and Feeding Preference

Piglets fed HP had greater final body weight (FBW) and average daily gain (ADG) than those fed SBM or RSB (*p* = 0.0124), whereas HP did not differ significantly from FSBM or ESB. Piglets fed FSBM also had greater FBW and ADG than those fed SBM (Table 3).

### 3.2. Feeding Preference

No significant differences were observed in IBW and FBW among the SBM, RSB, FSBM, HP, and ESB groups (*p* > 0.05). Meanwhile, no significant differences in FP and FPI were detected among the RSB, FSBM, HP, and ESB groups (*p* > 0.05) (Table 4).

### 3.3. Crude Protein Digestibility

**Figure 1:**
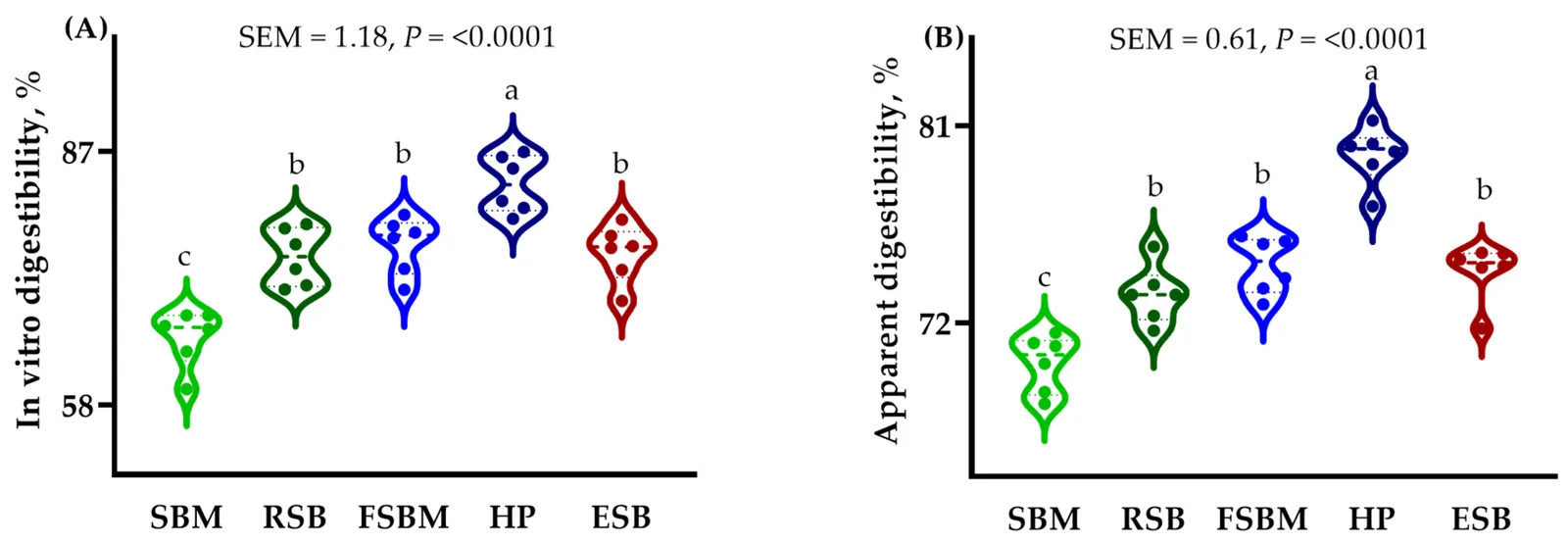
Effects of processed soybean proteins on crude protein digestibility in weaned piglets. (**A**) Mean in vitro crude protein digestibility was 65.69%, 74.93%, 76.30%, 83.25%, and 75.33% in the SBM, RSB, FSBM, HP, and ESB groups, respectively. (**B**) Mean apparent total-tract crude protein digestibility was 70.15%, 73.29%, 74.63%, 79.64%, and 74.35%, respectively. Means with different lowercase letters differ significantly (*p* < 0.05). SBM, soybean meal; RSB, rapidly expanded soybean meal; FSBM, fermented soybean meal; HP, hydrolyzed soybean protein; ESB, extruded full-fat soybean; SEM, standard error of the mean.

Soybean processing affected crude protein digestibility (*p* < 0.0001). HP had the highest in vitro and apparent total-tract crude protein digestibility among the five treatments (*p* < 0.0001; Figure 1).

### 3.4. Serum Immune Indices

For immune indices, serum IgA concentration in the HP group was significantly elevated compared with the SBM, RSB, FSBM and ESB groups, the ESB group was significantly higher than the SBM and RSB groups, and the RSB group was significantly higher than the SBM group (*p* < 0.0001).

For inflammatory cytokines, serum IL-2 in the HP group was significantly higher than that in the SBM, RSB and FSBM groups, and the ESB group was significantly higher than the SBM group (*p* = 0.0073). Serum TNF-α in the HP group was significantly reduced compared with the SBM, RSB, FSBM and ESB groups, whereas the FSBM and ESB groups had lower concentrations than the SBM and RSB groups but higher concentrations than the HP group (*p* < 0.0001) (Table 5).

### 3.5. Serum Antioxidant Indices

Serum GSH-Px, SOD, CAT, T-AOC, and MDA did not differ significantly among treatments (*p* > 0.05; Table 6).

### 3.6. Serum Hormone Levels

Serum GH in the HP group was significantly higher than that in the SBM, RSB, FSBM and ESB groups, and the RSB and FSBM groups were significantly higher than the SBM group; ESB did not differ significantly from SBM, RSB, or FSBM (*p* < 0.0001). Ghr levels in the FSBM, HP and ESB groups were higher than those in the SBM and RSB groups (*p* = 0.0316). Lep and CCK levels in the RSB, FSBM, HP and ESB groups were significantly lower than those in the SBM group, and the FSBM, HP and ESB groups were significantly lower than the RSB group (*p* = 0.0365; *p* = 0.0452). IGF-1 levels in the HP group were significantly elevated compared with the SBM, RSB, FSBM and ESB groups, while the RSB, FSBM, and ESB groups were significantly higher than the SBM group (*p* < 0.0001). (Table 7).

### 3.7. Serum Biochemical Indices

Serum TP levels in the RSB, FSBM, HP and ESB groups were significantly higher than those in the SBM group (*p* = 0.0012). However, serum TG levels in the RSB, FSBM, HP and ESB groups were significantly lower than those in the SBM group (*p* = 0.0030). Serum BUN levels in the FSBM, HP and ESB groups were significantly lower than those in the SBM and RSB groups (*p* < 0.0001). Serum HDL levels in the RSB, FSBM, HP and ESB groups were significantly higher than those in the SBM group (*p* = 0.0015). Serum LDL levels in the FSBM, HP and ESB groups were significantly lower than those in the SBM and RSB groups, and the FSBM, HP and ESB groups were significantly lower than the RSB group (*p* = 0.0215) (Table 8).

### 3.8. Analysis of Colonic Microbial Alpha Diversity

Alpha diversity was evaluated using Sobs, Chao1, ACE, Shannon, Simpson, Good’s coverage, and Faith’s phylogenetic diversity. Sobs represents the observed number of ASVs; Chao1 and ACE estimate richness; Shannon and Simpson describe diversity and evenness; Good’s coverage estimates sampling completeness; and Faith’s phylogenetic diversity incorporates branch length.

Good’s coverage exceeded 0.99 in all treatment groups, indicating high sampling coverage. Sobs (*p* = 0.9991), Chao1 (*p* = 0.9472), ACE (*p* = 0.9997), Shannon (*p* = 0.9993), and Simpson (*p* = 0.0829) did not differ significantly among treatments. Thus, the tested soybean protein sources were not associated with statistically significant differences in the measured alpha-diversity indices (Table 9).

**Figure 2:**
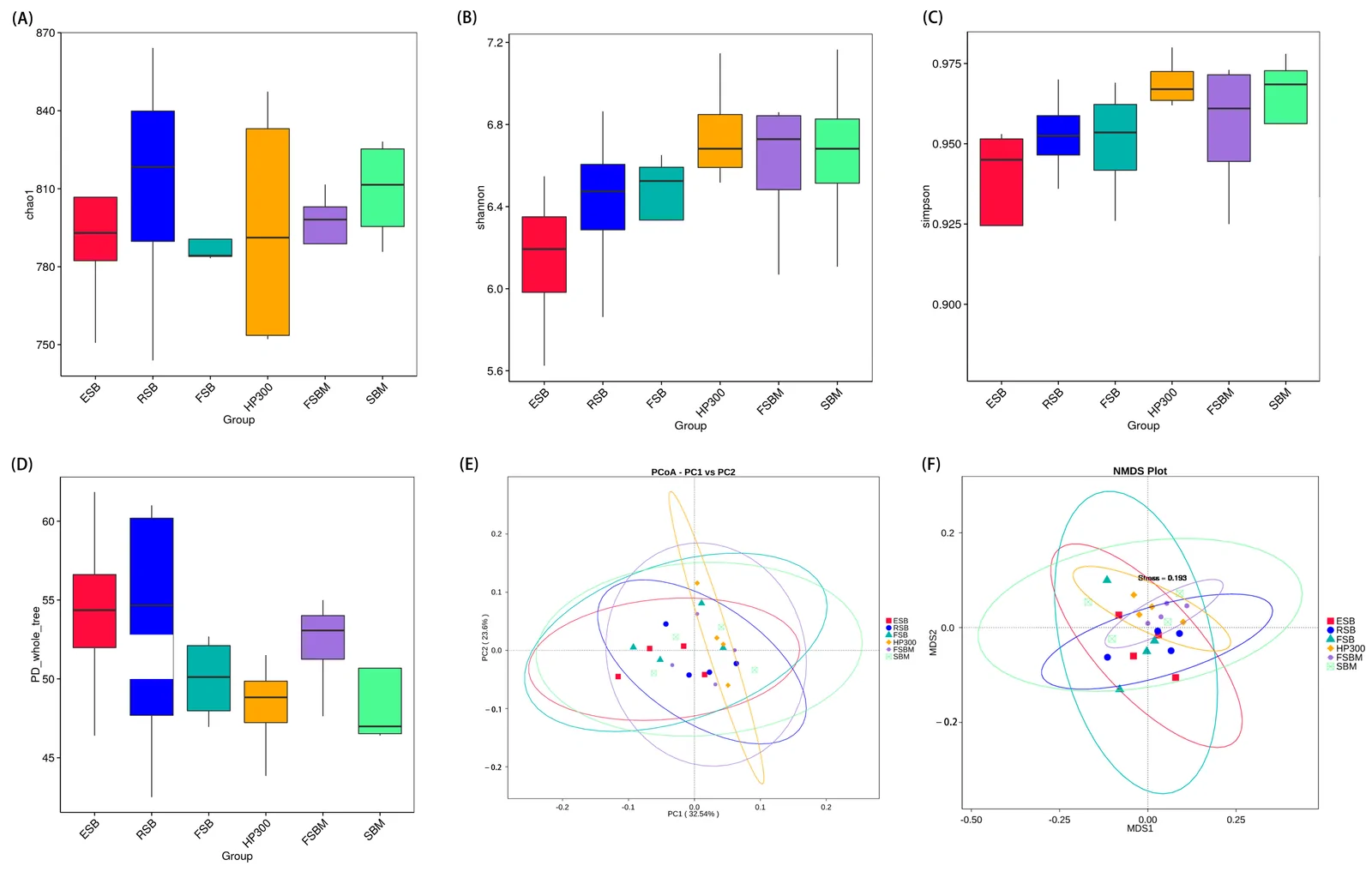
Alpha- and beta-diversity analyses of colonic microbial communities. (**A**) Chao1 index; (**B**) Shannon index; (**C**) Simpson index; (**D**) Faith’s phylogenetic diversity; (**E**) weighted UniFrac principal coordinate analysis (PCoA), with axes 1 and 2 explaining 32.54% and 23.60% of the variation, respectively; and (**F**) nonmetric multidimensional scaling (NMDS). Ordination panels are descriptive and do not demonstrate statistically significant group separation. SBM, soybean meal; RSB, rapidly expanded soybean meal; FSBM, fermented soybean meal; HP, hydrolyzed soybean protein; ESB, extruded full-fat soybean.

Alpha-diversity indices showed numerical variation among treatments, but none differed significantly (Table 9; Figure 2A–D). Weighted UniFrac PCoA explained 32.54% and 23.60% of the variation on the first two axes, respectively, and NMDS had a stress value of 0.193 (Figure 2E,F). These ordinations provide descriptive visualization only. Because a valid PERMANOVA output was unavailable, the apparent spatial separation cannot be interpreted as a statistically significant treatment effect.

The PCoA and NMDS plots suggested directional differences in sample positions; however, ordination does not test statistical significance. Given the small number of sequenced samples (*n* = 4 per treatment) and the absence of a valid PERMANOVA result, these patterns should be regarded as exploratory.

### 3.9. Analysis of Colonic Microbiota Composition

**Figure 3:**
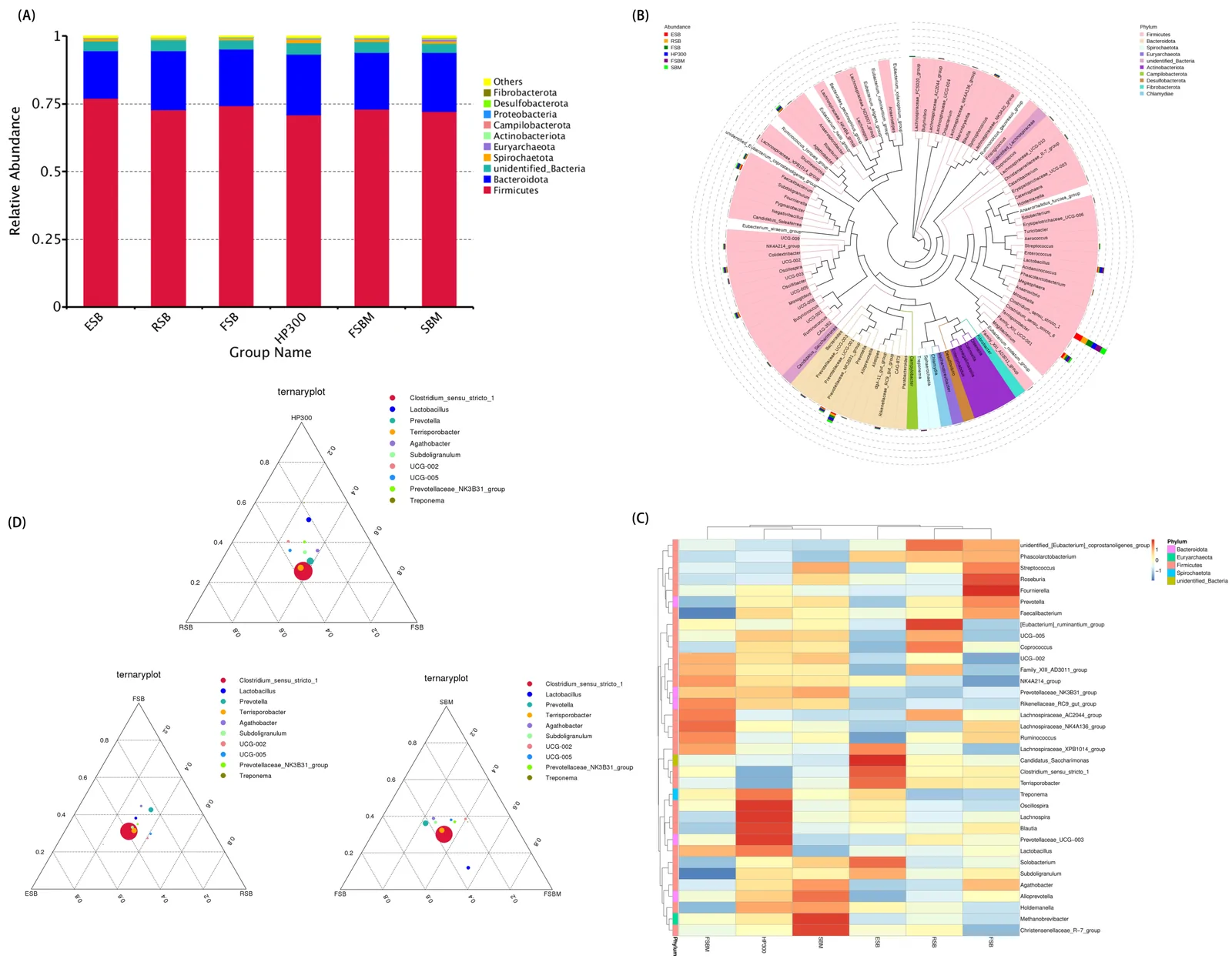
Composition and exploratory variation in colonic microbial communities. (**A**) Relative abundance at the phylum level; (**B**) circular phylogenetic tree of the 100 most abundant genera; (**C**) hierarchical clustering heatmap of genus-level relative abundance, shown as row-normalized Z scores; and (**D**) ternary plots showing the distribution of selected genera among treatment combinations. Dot size represents mean relative abundance. These panels are descriptive and do not by themselves establish statistically significant differential abundance. SBM, soybean meal; RSB, rapidly expanded soybean meal; FSBM, fermented soybean meal; HP, hydrolyzed soybean protein; ESB, extruded full-fat soybean.

At the phylum level, Firmicutes and Bacteroidota predominated across all groups, followed by Spirochaetota and Euryarchaeota (Figure 3A). The overall phylum-level profiles appeared broadly similar among treatments. The circular tree in Figure 3B depicts the phylogenetic relationships and relative-abundance patterns of the 100 most abundant genera; it is not a functional classification. Lactobacillus, Prevotella, and Clostridium_sensu_stricto_1 were among the predominant genera.

The genus-level heatmap showed treatment-associated patterns in relative abundance (Figure 3C). Christensenellaceae_R-7_group and Methanobrevibacter had higher relative abundance in the SBM samples, Lachnospira and Blautia in the RSB samples, Lactobacillus in the FSBM and HP samples, and Clostridium_sensu_stricto_1 in the ESB samples. Because these visualizations were exploratory and no multiple-comparison-adjusted differential-abundance test was available, the patterns should not be described as statistically significant enrichment.

The ternary plots display the observed distribution of individual taxa among selected treatment combinations (Figure 3D). They indicate relative-abundance patterns only and do not establish treatment-driven enrichment, microbial function, or causal relationships with host outcomes.

## 4. Discussion

Weaning combines dietary, environmental, and social changes that may suppress feed intake and compromise digestive and intestinal function [16,17]. Antinutritional and antigenic components of conventional soybean proteins can further challenge newly weaned piglets [18]. Processing is therefore used to modify protein structure and reduce these compounds [19,20]. Previous studies have frequently focused on growth, digestibility, or intestinal morphology separately [21,22]. The present study compared five commercial soybean protein sources within a common design and integrated growth, feeding preference, crude protein digestibility, serum biochemical, immune, antioxidant, and endocrine indices, and colonic microbiota.

HP contained no detectable glycinin and had the lowest measured β-conglycinin concentration among the tested ingredients, together with the highest in vitro and apparent crude protein digestibility. Piglets fed HP also had greater ADG and FBW than those fed SBM or RSB. These concurrent observations are consistent with earlier reports on enzyme-treated soybean products [23,24,25], but they do not prove that allergen reduction caused the growth response. Intact glycinin and β-conglycinin can impair intestinal function in weaned piglets [26], and enzymatic hydrolysis can reduce their antigenicity [27,28].

Piglets fed HP had higher serum IgA and IL-2 concentrations and lower serum TNF-α concentrations than those fed SBM. Previous studies suggest that residual soybean antigenic proteins may influence inflammatory signaling in weaned piglets [29,30,31]. The lower serum TNF-α concentration in the HP group was therefore consistent with, but did not establish, reduced inflammatory signaling. Because intestinal morphology, permeability, mucosal IgA, tight-junction proteins, and the phosphorylated forms of p38, JNK, and NF-κB p65 were not measured, the present data do not demonstrate repair or reinforcement of the physical or immune intestinal barrier.

FSBM and RSB had higher crude protein digestibility than SBM, but their growth responses were smaller than that of HP. Table 1 provides a compositional context: FSBM retained 12.09 mg/g glycinin and 10.56 mg/g β-conglycinin, whereas glycinin was not detected in HP and β-conglycinin was 1.79 mg/g; HP also had a higher crude protein concentration (56.18% vs. 48.24%). RSB retained higher concentrations of the measured antigenic proteins and had a lower crude protein concentration than HP. These differences may partly explain the observed response patterns, but causality cannot be assigned because processing conditions and peptide profiles were not fully characterized and the ingredient inclusion rates differed among diets. Feed preference did not differ among treatments, indicating that the growth differences were unlikely to be explained solely by the measured preference outcomes. Differences among commercial FSBM studies may also reflect processing conditions, microbial strains, ingredient composition, diet formulation, and experimental duration [32,33,34,35,36,37].

Piglets fed HP had higher serum GH and IGF-1 and lower serum leptin and CCK concentrations than those fed SBM, while serum ghrelin was higher than in the SBM and RSB groups. These patterns are consistent with differences in circulating endocrine indicators related to growth and nutrient status [38,39,40,41]. However, circulating hormone concentrations alone do not demonstrate activation of the complete GH–IGF-1 pathway or increased protein deposition. Similarly, lower BUN may reflect altered nitrogen metabolism but is not direct evidence of tissue protein accretion [42]. Differences in serum HDL and LDL describe circulating lipoprotein profiles but do not demonstrate altered lipid deposition, adiposity, or body composition.

Serum antioxidant indicators did not differ among treatments under the conditions of this trial. Because the study was conducted without an intentional environmental or pathogenic challenge, the results cannot establish whether the soybean protein sources would produce different antioxidant responses under stress. The absence of significant differences should therefore be interpreted as a lack of detectable treatment effects on the measured serum antioxidant indices in the present experimental setting, rather than evidence of equivalent biological antioxidant capacity.

Microbial alpha-diversity indices did not differ significantly among treatments. Descriptive relative-abundance profiles showed numerically higher Lactobacillus abundance in the HP and FSBM samples and other treatment-associated patterns for selected genera. However, because the microbiota analysis included only four samples per treatment and no multiple-comparison-adjusted differential-abundance test was available, these observations cannot establish treatment-driven enrichment. Functional characteristics previously attributed to Lactobacillus, Prevotella, and Clostridium in the literature cannot be inferred directly from the present 16S rRNA gene sequencing data and require confirmation using adequately powered metagenomic, transcriptomic, metabolomic, or targeted intervention studies.

Several considerations should guide interpretation of these findings. The 28-day trials evaluated short-term nutritional responses during the nursery phase and cannot establish longer-term effects. Although the diets were formulated to be similar in energy, crude protein, and major digestible amino acids, residual differences in crude fat, amino acid concentrations, ingredient inclusion rates, and uncharacterized peptide profiles may have contributed to the observed responses. The microbiota analysis included only four samples per treatment and relied on 16S rRNA gene sequencing; therefore, genus-level relative-abundance patterns should be regarded as exploratory and cannot establish microbial function or causal relationships with host outcomes. In addition, serum immune, metabolic, and endocrine indices do not by themselves demonstrate intestinal barrier repair, tissue protein deposition, or activation of specific signaling pathways. The use of Tunchang Black piglets and commercial products from single manufacturing sources may limit generalizability to commercial crossbred pigs and other product batches. Future studies should use longer trials, independently manufactured product batches, and commercial genotypes, together with intestinal morphology and permeability measurements, nitrogen balance, tissue composition, targeted endocrine signaling, shotgun metagenomics, and metabolomics. Adequately powered microbiota analyses should prespecify sequence-processing parameters, PERMANOVA, and false-discovery-rate control.

## 5. Conclusions

**Figure 4:**
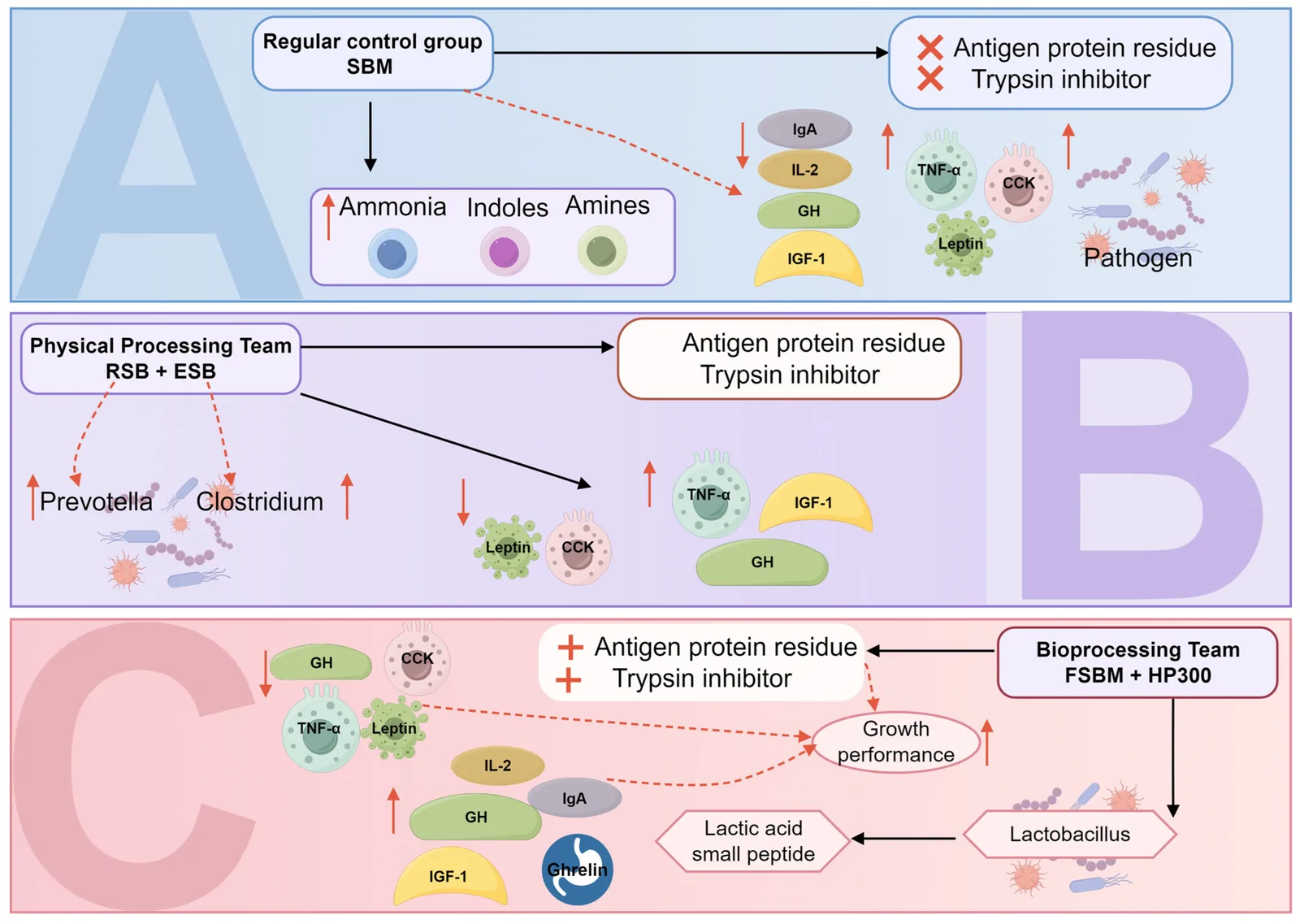
Comparative effects of processed soybean protein sources in weaned piglets. (**A**) Conventional soybean meal control group; (**B**) Physical processing group, including rapidly expanded soybean meal and extruded full-fat soybean; (**C**) Bioprocessing group, including fermented soybean meal and hydrolyzed soybean protein. Solid statements summarize measured outcomes; proposed mechanisms should be interpreted as hypotheses unless directly tested. SBM, soybean meal; RSB, rapidly expanded soybean meal; FSBM, fermented soybean meal; HP, hydrolyzed soybean protein; ESB, extruded full-fat soybean.

This study compared five processed soybean protein sources using growth, feed preference, crude protein digestibility, serum indices, and colonic microbiota (Figure 4). HP contained the lowest concentrations of the measured antigenic proteins, had the highest crude protein digestibility, and was associated with greater ADG and FBW than SBM and RSB. HP also altered selected immune and endocrine serum indices. Feed preference and serum antioxidant indices did not differ among treatments. Microbial alpha diversity was similar across groups, and genus-level relative-abundance patterns were exploratory because of the small sequencing sample size and incomplete inferential analysis. The study therefore provides comparative evidence for ingredient selection but does not establish intestinal barrier repair, microbial function, protein deposition, or activation of specific molecular pathways. Validation in commercial genotypes, additional product batches, and longer trials is required.

## Figures and Tables

**Table 1:** Nutritional composition and antinutritional factor concentrations (analytical values) of differently processed soybean protein.

Items	SBM	RSB	FSBM	HP	ESB
Dry matter, %	88.23	90.15	89.12	92.26	89.55
Crude protein, %	45.62	36.19	48.24	56.18	36.25
Ether extract, %	1.19	20.57	1.56	0.97	19.86
Trypsin inhibitor, mg/g	5.72	1.20	0.54	0.82	2.57
Glycinin, mg/g	146.23	33.62	12.09	- ^1^	32.24
β-Conglycinin, mg/g	136.19	22.64	10.56	1.79	18.69

^1^ Not detected; SBM, Soybean meal; RSB, rapidly expanded soybean meal; FSBM, Fermented soybean meal; HP, hydrolyzed soybean protein; ESB, Extruded full-fat soybean meal.

**Table 2:** Ingredient composition and nutritional levels of basal experimental diets (air-dry basis, %).

Items	SBM	RSB	FSBM	HP	ESB
Raw materials					
Corn	10	10	10	10	10
Extruded corn	53.74	53.14	54.34	57.54	53.14
SBM	25.18	13.58	13.58	13.58	13.58
ESB					15
RSB		15			
FSBM			11		
HP				9	
Fish meal	1	1	1	1	1
Soybean oil	3.3	0.5	3.3	2.1	0.5
White sugar	2.2	2.2	2.2	2.2	2.2
Choline chloride	0.1	0.1	0.1	0.1	0.1
Dicalcium phosphate	0.7	0.7	0.7	0.7	0.7
Calcium formate	0.6	0.6	0.6	0.6	0.6
Salt	0.25	0.25	0.25	0.25	0.25
Lysine	0.44	0.44	0.44	0.44	0.44
Methionine	0.12	0.12	0.12	0.12	0.12
Threonine	0.19	0.19	0.19	0.19	0.19
Zinc oxide	0.18	0.18	0.18	0.18	0.18
Wheat middlings	1	1	1	1	1
Premix ^1^	1	1	1	1	1
Total	100.00	100.00	100.00	100.00	100.00
Nutrient Levels					
Digestible energy ^2^, MJ/kg	14.20	14.18	14.10	14.17	14.15
Crude protein ^3^, %	17.62	17.52	17.60	17.50	17.35
Crude fat ^3^	5.75	6.02	6.10	5.20	5.78
Lysine ^3^	1.17	1.21	1.21	1.19	1.21
Methionine ^3^	0.39	0.39	0.39	0.40	0.39
Tryptophan ^3^	0.22	0.23	0.23	0.28	0.23
Calcium ^2^	0.76	0.76	0.76	0.76	0.76
Total phosphorus ^2^	0.62	0.62	0.62	0.62	0.62
Available phosphorus ^2^	0.40	0.40	0.40	0.40	0.40

^1^ Premix can provide per kg of full-price ration: Vitamin A 7 500 IU, Vitamin D_3_ 750 IU, Vitamin E 25 IU, Vitamin K_3_ 2.0 mg, Vitamin B_1_ 1.88 mg, Vitamin B_2_ 3.75 mg, Vitamin B_6_ 2.19 mg, Vitamin B_12_ 0.025 mg, Niacin 25 mg, Pantothenic acid 15.6 mg, Folic acid 2.0 mg, Biotin 0.188 mg; Copper 25 mg, Zinc 100 mg, Iron 100 mg, Manganese 30 mg, Selenium 0.3 mg, Iodine 0.4 mg. ^2^ Calculated value. ^3^ Analyzed value. SBM, Soybean meal; RSB, Rapidly expanded soybean meal; FSBM, Fermented soybean meal; HP, Hydrolyzed soybean protein; ESB, Extruded full-fat soybean meal.

**Table 3:** Effects of differently processed soybean protein on growth performance of weaned piglets.

Items	SBM	RSB	FSBM	HP	ESB	SEM	*p*-Value
IBW, kg	5.87	5.87	5.86	5.86	5.87	0.11	0.9999
FBW, kg	14.28 ^c^	14.37 ^bc^	14.56 ^ab^	14.68 ^a^	14.51 ^abc^	0.04	0.0124
ADFI, g/d	460.84	465.32	463.84	458.24	467.11	5.07	0.9759
ADG, g/d	300.50 ^c^	303.50 ^bc^	310.50 ^ab^	315.01 ^a^	308.50 ^abc^	1.53	0.0124
FCR	1.54	1.53	1.49	1.46	1.51	0.01	0.3908

IBW, initial body weight; FBW, final body weight; ADFI, average daily feed intake; ADG, average daily gain; FCR, feed conversion ratio; SBM, soybean meal; RSB, rapidly expanded soybean meal; FSBM, fermented soybean meal; HP, hydrolyzed soybean protein; ESB, extruded full-fat soybean; SEM, standard error of the mean. Within each row, means with different lowercase superscript letters differ significantly (*p* < 0.05).

**Table 4:** Effects of differently processed soybean protein on feed preference of weaned piglets.

Items	SBM	RSB	FSBM	HP	ESB	SEM	*p*-Value
IBW, kg	5.86	5.86	5.87	5.87	5.87	0.12	0.9832
FBW, kg	14.18	14.19	14.20	14.22	14.21	0.20	0.1326
FP, %	/	62.83	61.39	61.69	61.39	1.04	0.7035
FPI	/	1.62	1.59	1.61	1.59	0.02	0.9201

IBW, initial body weight; FBW, final body weight; FP, feed preference; FPI, feed preference index; SBM, soybean meal; RSB, rapidly expanded soybean meal; FSBM, fermented soybean meal; HP, hydrolyzed soybean protein; ESB, extruded full-fat soybean; SEM, standard error of the mean.

**Table 5:** Effects of differently processed soybean protein on serum immune indices of weaned piglets.

Items	SBM	RSB	FSBM	HP	ESB	SEM	*p*-Value
IgA, g/L	1.50 ^d^	1.57 ^c^	1.59 ^bc^	1.72 ^a^	1.61 ^b^	0.01	<0.0001
IgG, g/L	7.33	7.33	7.29	7.35	7.30	0.03	0.9001
IgM, g/L	0.87	0.87	0.85	0.87	0.86	0.02	0.9987
IL-2, pg/mL	162.15 ^bc^	161.15 ^bc^	160.12 ^c^	168.35 ^a^	165.37 ^ab^	0.87	0.0073
TNF-α, ng/L	80.25 ^a^	80.31 ^a^	75.31 ^b^	70.30 ^c^	76.39 ^b^	0.78	<0.0001

IgA, immunoglobulin A; IgG, immunoglobulin G; IgM, immunoglobulin M; IL-2, interleukin-2; TNF-α, tumor necrosis factor-α; SBM, soybean meal; RSB, rapidly expanded soybean meal; FSBM, fermented soybean meal; HP, hydrolyzed soybean protein; ESB, extruded full-fat soybean; SEM, standard error of the mean. Within each row, means with different lowercase superscript letters differ significantly (*p* < 0.05).

**Table 6:** Effects of differently processed soybean protein on serum antioxidant indices of weaned piglets.

Items	SBM	RSB	FSBM	HP	ESB	SEM	*p*-Value
GSH-Px, U/mL	157.37	152.91	152.36	151.33	155.48	3.59	0.1643
SOD, U/mL	105.33	107.92	112.39	100.55	100.29	1.80	0.1604
CAT, U/mL	10.11	9.79	10.13	10.29	9.92	0.25	0.9800
T-AOC, U/mL	15.31	15.02	14.92	15.33	14.99	0.36	0.9948
MDA, nmol/mL	4.93	5.00	4.92	5.02	5.03	0.15	0.9992

GSH-Px, glutathione peroxidase; SOD, superoxide dismutase; CAT, catalase; T-AOC, total antioxidant capacity; MDA, malondialdehyde; SBM, soybean meal; RSB, rapidly expanded soybean meal; FSBM, fermented soybean meal; HP, hydrolyzed soybean protein; ESB, extruded full-fat soybean; SEM, standard error of the mean.

**Table 7:** Effects of differently processed soybean protein on serum hormone indices of weaned piglets.

Items	SBM	RSB	FSBM	HP	ESB	SEM	*p*-Value
GH, ng/mL	8.01 ^c^	8.46 ^b^	8.52 ^b^	9.56 ^a^	8.33 ^bc^	0.11	<0.0001
Ghr, pg/mL	236.77 ^b^	240.25 ^b^	265.12 ^a^	267.55 ^a^	266.39 ^a^	2.70	0.0316
Lep, ng/mL	4.33 ^a^	4.15 ^b^	3.57 ^c^	3.56 ^c^	3.52 ^c^	0.07	0.0365
CCK, pg/mL	75.33 ^a^	67.56 ^b^	51.14 ^c^	50.12 ^c^	52.19 ^c^	1.97	0.0452
IGF-1, ng/mL	231.29 ^c^	245.26 ^b^	246.37 ^b^	267.53 ^a^	250.11 ^b^	2.26	<0.0001

GH, growth hormone; Ghr, ghrelin; Lep, leptin; CCK, cholecystokinin; IGF-1, insulin-like growth factor-1; SBM, soybean meal; RSB, rapidly expanded soybean meal; FSBM, fermented soybean meal; HP, hydrolyzed soybean protein; ESB, extruded full-fat soybean; SEM, standard error of the mean. Within each row, means with different lowercase superscript letters differ significantly (*p* < 0.05).

**Table 8:** Effects of differently processed soybean protein on serum biochemical indices of weaned piglets.

Items	SBM	RSB	FSBM	HP	ESB	SEM	*p*-Value
TP, g/L	70.15 ^b^	76.31 ^a^	76.36 ^a^	75.32 ^a^	75.59 ^a^	0.48	0.0012
ALB, g/L	40.12	39.57	41.33	40.56	40.92	0.41	0.7180
GLB, g/L	29.57	29.63	30.11	29.97	30.51	0.38	0.9448
TG, mmol/L	0.73 ^a^	0.63 ^b^	0.63 ^b^	0.61 ^b^	0.63 ^b^	0.01	0.0030
TC, mmol/L	2.01	2.13	2.13	2.02	2.02	0.14	0.9977
BUN, mmol/L	4.57 ^a^	4.53 ^a^	3.15 ^b^	3.13 ^b^	3.17 ^b^	0.13	<0.0001
HDL, mmol/L	0.91 ^b^	1.31 ^a^	1.46 ^a^	1.47 ^a^	1.43 ^a^	0.04	0.0015
LDL, mmol/L	1.65 ^a^	1.41 ^b^	1.23 ^c^	1.22 ^c^	1.19 ^c^	0.04	0.0215

TP, total protein; ALB, albumin; GLB, globulin; TG, triglycerides; TC, total cholesterol; BUN, blood urea nitrogen; HDL, high-density lipoprotein; LDL, low-density lipoprotein; SBM, soybean meal; RSB, rapidly expanded soybean meal; FSBM, fermented soybean meal; HP, hydrolyzed soybean protein; ESB, extruded full-fat soybean; SEM, standard error of the mean. Within each row, means with different lowercase superscript letters differ significantly (*p* < 0.05).

**Table 9:** Effects of differently processed soybean protein on colonic microbial alpha diversity of weaned piglets.

Items	SBM	RSB	FSBM	HP	ESB	SEM	*p*-Value
Sobs	750	748	747	750	751	5.65	0.9991
Chao1	792	795	800	810	806	7.44	0.9472
Ace	789	790	792	791	795	8.50	0.9997
Shannon	6.54	6.53	6.49	6.53	6.52	0.11	0.9993
Simpson	0.93	0.92	0.93	0.95	0.95	0.00	0.0829
Coverage	0.998	0.998	0.998	0.998	0.998	/	/
Faith’s PD	6.875	6.922	6.786	7.050	6.837	0.12	0.3256

SBM, Soybean meal; RSB, Rapidly expanded soybean meal; FSBM, Fermented soybean meal; HP, Hydrolyzed soybean protein; ESB, Extruded full-fat soybean meal. SEM, Standard error of the mean.

## Data Availability

Sequence data have been deposited in the NCBI Sequence Read Archive under accession PRJNA1478974.
